# Supplementary material for: Evaluation of knowledge, attitudes, and clinical education of dental students about COVID-19 pandemic
Source: PeerJ. 2020 Jul 29;8:e9575. doi: 10.7717/peerj.9575 (PMC7395615; doi:10.7717/peerj.9575)
Supplement: Supplemental Information 2 [file peerj-08-9575-s002.docx]

Demographic data

**Gender**  Male Female

**Course period** Clinic Preclinic

**Clinical Rotations**

Oral and Maxillofacial Surgery

Endodontics

Periodontology

Prosthodontic

Oral Diagnosis and Radiology

Restorative dentistry

Pediatric Dentistry

Orthodontics

**Have you been informed about COVID-19 in your faculty like lectures or seminars?**

Yes No

**Have you asked questions such as high fever, dry cough or travel abroad while taking an anamnesis from your patients in the last 3 months?**

Yes No

**Did COVID-19 negatively affect your psychology?**

Yes No Undecided

**Are you afraid of becoming infected with COVID-19 as a healthcare professional working at close range with the patient?**

Yes No Undecided

**Are you afraid to infect any relatives or people around you in terms of COVID-19 because you are a healthcare worker working very close to the patient?**

Yes No Undecided

**Would you hesitate to treat a patient who came to dental treatment after getting over and recovering from COVID-19 infection?**

Yes No Undecided

**Do you think that after your COVID-19 pandemic, you will be more careful in your standard measures regarding contamination in your patients?**

Yes No Undecided

**With the COVID-19 outbreak, did you regret that you chose the dentistry profession?**

Yes No Undecided

**Do antibiotics benefit in the treatment of COVID- 19?**

Yes No I don’t know

**Can a mother diagnosed with COVID-19 breastfeed her child?**

Yes No I don’t know

**Individual measures taken by our students against COVID -19 in their daily lives**

Gloves

Mask

Frequent hand-washing

Use of cologne, wet wipes, and hand disinfectant

Not being in crowded places

Not having physical contact (handshaking, kissing, etc.)

Frequent ventilation for a healthy indoor environment

Changing clothes

Taking a shower upon arrival home

None

**Measures taken by clinical students for themselves in clinical rotation related to COVID-19**

Gloves

Mask

FF3/N95 mask

Face protective shield

Goggles

Bonnet

Disposable apron

Frequent hand-washing

Frequent hand sanitizing

**Precautions taken with the patient regarding COVID-19 while treating teeth**

**Before a dental procedure, I ask patients if they have symptoms such as a fever and a cough**

I measured the patient’s fever

I’m applying a rubber dam

Before a dental procedure, I have patients rinse their mouth with an antiseptic mouthwash containing chlorhexidine

Before a dental procedure, I have patients rinse their mouth with an antiseptic mouthwash containing 1% hydrogen peroxide,

I use a strong absorbent system during the process

I avoid all the procedures that will create an aerosol as much as possible

I prefer to use hand tools instead of an aerator, cavitron, or micromotor,

I apply the least 14-day waiting rule to potentially infected patients

None

**Sources about information about COVID-19**

Websites or social media accounts of professional organizations, such as the Ministry of Health, Dental Association, and WHO,

Events such as seminars/ meetings held by institutions

Published scientific articles,

Physicians’ individual websites or social media accounts,

Social media accounts, like Instagram, Facebook, and Twitter,

Television and radio programs,

Communication groups, such as Whatsapp or Line
